# Remarkable Case of Gunshot Wound of the Bladder, Requiring Two Operations of Lithotomy, One Seven and the Other Eleven Months after Receipt of the Injury, and an Operation for Fistula in Peineo, Six Months Later: Recovery Perfect

**Published:** 1866-10

**Authors:** Carlisle Terry

**Affiliations:** Columbus, Georgia


*Remarkable Case of Gunshot Wound of the Bladder, requiring two Operations of Lithotomy, one seven and the other eleven Months after receipt of the Injury, and an Operation for Fistula in Peineo, six months later: Recovery perfect.* By CARLISLE TERRY, M. D., Columbus, Georgia.

J. A. M., Co. E., 39th Alabama Regiment, was wounded on the 28th of July, 1864, in battle near Atlanta, by a minie ball passing through the sacrum near its left iliac junction, then transversing the pelvis, it passed through the walls of the bladder, and made its exit through the pubis to the right of the symphysis. He was admitted into hospital in Atlanta, and remained there fifteen days. While there the anterior wound healed. About that time an abscess formed above the anterior wound, near the anterio-inferior spinous process. He was admitted into Walker Hospital, Columbus, Georgia, under my charge, on the 5th of September, 1864.

The urine was then passing partly from the posterior wound. Soon after, several abscesses formed near the wound of exit, close to the right side of the scrotum, involving the right testicle, most of which sloughed out, probably on account of injury to spermatic vessels. The posterior wound did not heal, until three months after the receipt of injury. About that time, the abcesses around the root of scrotum also healed, yet the fistula near the anterio-inferior process remained open, through which was discharged a portion of the urine; also several small pieces of bone. Small pieces of bone and several urinary calculi also passed through the urethra during this time.

The suffering experienced during micturition, and the pain in pelvis, induced me to explore the urinary canal with a sound. On passing the instrument into the membranous portion of the urethra, a grating sensation was felt. The diagnosis having been made now certain by subsequent examinations, an operation was determined upon for the removal of the foreign body.

On the 3d of March, 1865, the patient was placed on the table, and a straight staff passed into the urethra down to the foreign body. It had been impossible to pass a sound or catheter into the bladder, or to any distance below this. The cause was disclosed as the operation proceeded. Upon opening the urethra, a piece of bone one and one-half inches long, was found lying transversely across the urethra and imbedded in the muscles. This was found to be covered with calcareous matter, and was, after a little manipulation, removed with the forceps. Further examination then disclosed a number of pieces of bone and a large amount of calcareous matter in the membranous portion of the urethra, which was found to be much elongated and attached to the pelvis, making a short angle around it, thus explaining why no instrument could be passed into the bladder. The urethra from this point was found to be much enlarged, elongated and attached to the inner face of the pubis, the prostate being at the upper end of the symphysis. The finger being used as a director, the remainder of the urethra was carefully opened with a bistoury; many pieces of bone and a good deal of calcareous matter removed. Upon reaching the prostate, the point of the finger could be nearly passed into the bladder. The gland was sufficiently incised with the bistoury, and the finger passed into the bladder, in which several more pieces of bone were found and more calcareous matter. One piece, about the size of a filbert, was found enclosed in a cyst, which, however, was thin enough to be opened with the finger nail. The bladder was then well washed out with copious injections of warm water. So much of the bone was in very small pieces, and the calcareous matter being so soft, a large portion of it was lost, having become mixed with the clots and washed away in sponging. It was thought that about as much as would fill the palm of the hand was removed.

The patient suffered with considerable fever for the next three days, which subsiding, he continued to improve, though much irritated by the passage through the wound of small pieces of bone and sand-like deposits.

On the eighth day after the operation, he had high fever, with severe headache, which continued three days, during which time the urine commenced to flow through the fistula, near the right anterior spinous process, in consequence of which the catheter was introduced into the bladder, through the wound. About the same time he suffered from inflammatory engorgement of the scrotum.

The patient continued to improve for about one month. The incision healed and urine passed almost entirely through the urethra, except that occasionally the fistula would discharge a little of it. About this time calcareous matter began to be discharged, and increased irritation of the bladder gave evidence that more bone was making its way into it, and small spiculæ were discharged by the urethra. It was judged best to sustain the patient as well as possible, and wait in hopes that after all of the necrosed bone should have been thrown off, another operation might result in a final cure.

In April, the hospital having been broken up by the termination of the war, M. was removed to his father's, some twenty miles from the city. He continued to discharge a great deal of calcareous matter, and became so reduced by pain and irritation, that it was evident that he could hold out but little longer. On the 29th of June, I went down to see him; I found him very much emaciated and suffering all the time great pain and constant fever. I determined to operate at once, (though I had no medical assistance,) aided by his father and two neighbors.

The abrupt turn of the urethra around the pubis, and its attachment to the inner face of that bone, rendered the introduction of a sound to the bladder impossible. A staff was passed in as far as possible, and the usual lateral incision made to it, then a director readily passed from the wound through the remainder of the urethra to the bladder, and the stone was distinctly felt. With a probe-pointed bistoury the urethra was split up and the prostate incised. With the forceps several small pieces of bone and three calculi were extracted, the larger of which was about the size of a guinea's egg; the others the size of a hickory-nut. As at the first operation, there was a large amount of soft calcareous matter discharged with the urine, when the prostate was incised. After thoroughly washing out the bladder, an examination with the finger showed that the organ, so far as could be thus ascertained, was in a healthy condition, (which it was not after the first operation,) and gave reason to hope that the relief would be permanent.

The patient bore the operation well, and passed a comfortable night. I left the next morning, leaving a note requesting my friend, Dr. Geo. Heard, who lived five miles distant, to look after the patient, which he kindly did.

M. recovered rapidly, and with no bad symptoms. The fistula in the groin now quickly healed, but unfortunately the incision failed to heal, and except when closed by the finger, nearly all of the urine passed through it. Dr. Heard made several attempts to close it by cauterizing, but as he could not pass a catheter into the bladder through the penis, they were all unsuccessful.

In December, I had M. brought to town. He was then in health, and had passed no calcareous matter since the operation in June, but a large fistula remained in the perineum, through which all the urine escaped.

I first made an attempt to use a gum elastic catheter, which I could introduce readily into the bladder, by passing it through the penis to the fistula, and then with a strong director bending it, and guiding it into the bladder. I soon found that this would not answer, as in consequence of the abrupt bend in it, it soon became softened by the urine, and losing its elasticity, would become impervious, and the urine would discharge around it through the fistula as before. I then had a silver catheter prepared by bending it very short, and by careful manipulation I succeeded in introducing it into the bladder. Then by scarifying, cauterizing and stuffing the fistula with charpie, I obtained granulation from the bottom, and in six weeks the fistula was entirely closed and the catheter removed.

After nearly eighteen months' confinement to his bed, M. began to walk about and rapidly regained his health.

Upon resuming the erect position, however, he found that he could retain no urine, but that it ran guttatim as rapidly as secreted. Very gradually, but steadily, he regained power to retain it, until at this time, April 26, 1866, he can retain it to the amount of four ounces, when, unless voided, it will commence to drop. As his improvement in this respect has been so steady, it is to be presumed that in time he will be able to retain the usual quantity.

My friend, Mr. W. J. Land, an accomplished chemist of this city, has kindly made an analysis of the calculi, and finds them to consist almost entirely of phosphate of lime, with very slight traces of oxalate of lime, developed upon several small pieces of bone.

The operation on the 29th of June was contrary to the established rule—" always feel the stone before you operate,"—but the impossibility of reaching the bladder, the history of the case, and the certain speedy death of the patient, unless relieved, justified me in it, and the result proved the correctness of my diagnosis. I doubt if the operation for stone was ever before performed, without medical assistants. Mine, in this case, were plain country farmers.

This case, in both operations, showed the advantages of a surgeon's accustoming himself to operate without gorgets, lithotomes, etc. In this case, the bistoury (the instrument I always use) was the only one which could have been used successfully.

The difficulty in getting a silver catheter in, to cure the fistula, can be but poorly shown in the report. The curve was made very short, making nearly one-third of a circle, and which almost any one would say would be impossible to have been introduced. It formed an angle of but little more than 45°.—*Richmond Med. Journal.*

